# Dendritic retraction, but not atrophy, is consistent in amyotrophic lateral sclerosis-comparison between Onuf’s neurons and other sacral motor neurons-

**DOI:** 10.1186/2051-5960-2-11

**Published:** 2014-01-27

**Authors:** Takahiro Takeda, Toshiki Uchihara, Yuki Nakayama, Ayako Nakamura, Shoichi Sasaki, Shinji Kakei, Shinichiro Uchiyama, Charles Duyckaerts, Mari Yoshida

**Affiliations:** 1 Laboratory of Structural Neuropathology, Tokyo Metropolitan Institute of Medical Science, 2-1-6 Kamikitazawa, Setagaya-ku, Tokyo 156-8506, Japan; 2 Laboratory of Nursing Research for Intractable Disease, Tokyo Metropolitan Institute of Medical Science, 2-1-6 Kamikitazawa, Setagaya-ku, Tokyo 156-8506, Japan; 3 Research Project for Motor Control, Tokyo Metropolitan Institute of Medical Science, 2-1-6 Kamikitazawa, Setagaya-ku, Tokyo 156-8506, Japan; 4 Department of Neurology, Tokyo Women’s Medical University, 8-1 Kawada-cho, Shinjuku-ku, Tokyo 162-8666, Japan; 5 Raymond Escourolle Laboratory of Neuropathology, La Salpêtrière Hospital, 47 Boulevard de l′Hôpital, 75651 Paris Cedex 13, France; 6 Department of Neuropathology, Aichi Medical University, 1-1 Yazakokarimata, Nagakute, Aichi 480-1195, Japan

**Keywords:** Amyotrophic lateral sclerosis, Onuf's nucleus, Dendrite, Anterior horn, TDP43

## Abstract

**Background:**

Fundamental cytological changes of amyotrophic lateral sclerosis (ALS) were looked for by comparing relatively preserved Onuf’s nucleus (ON) and severely affected neighboring motor neuron groups (dorsolateral alpha motoneurons (DL) and other anterior horn neurons (OAH)). The second sacral segments from 11 ALS patients and 5 controls were initially quadruple-labeled for phosphorylated and non-phosphorylated TAR DNA-binding protein of 43 kDa (TDP43), and p62 with DAPI to identify TDP43-related changes. After digital recording of these fluorescence data encompassing the entire specimen at a high resolution, the same sections were stained with Klüver-Barrera method to obtain their exact bright-field counterparts. This novel approach facilitated exact identification of ON. Furthermore, this cell to cell comparison enabled to correlate quantitative indices of the neuronal cell bodies: perimeter, area and circularity index (CI) i.e. the ratio of (perimeter/2π) divided by the square root of (area/π), which decreases with dendritic retraction, overall number of neurons and inclusions.

**Results:**

In addition to known preservation of ON neuron number relative to DL and OAH, size reduction of ON neurons was not significant even in the advanced stage. Significant size reduction in DL was counteracted in the presence of TDP43-positive inclusions. Early increase of neuronal size in OAH was further enhanced by the presence of TDP43-positive inclusions. Even with these heterogeneous cytopathological changes, a decrease in CI was consistent in all groups at an early phase and was correlated with neuronal loss.

**Conclusions:**

Among variable cytological changes of ALS, a decrease in CI is a consistent early feature shared between non-atrophic ON neurons and other anterior horn neurons with either decreased (DL) or even increased (OAH) size and profounder neuronal loss. This decrease in CI, representative of dendritic retraction, is fundamental to ALS pathogenesis, not necessarily linked to cell size and pathological inclusions.

## Background

Relative preservation of Onuf’s nucleus (ON) at the sacral level of the spinal cord in amyotrophic lateral sclerosis (ALS) explains the sparing of the sphincter muscles, which is in sharp contrast with severe involvement of other upper and lower motor neurons [1]. Even though, the ON is not free from pathological changes: it has indeed been reported that neuronal numbers decrease, that neuronal atrophy with central chromatolysis is common [2,3], and that skein-like/rounded inclusions [2,4,5] or Bunina bodies [2,6] appear in the ON in ALS patients. However, only a few reports quantified neuronal loss, morphologic changes (shrinkage of cytoplasm and dendrites) and pathological inclusions. Moreover, mutual relationship between these quantified indices has never been reported in ALS. In the present study, we used a quadruple fluorescence method to correlate TAR-DNA binding protein of 43 kDa (TDP43) with or without phosphorylation and p62 epitopes and dislocation of nuclear TDP43 in individual neurons. Digital recording of the entire section by a virtual slide system at a high resolution enabled to capture all the pathological alterations of ALS, which are highly variable from a neuron to another. Subsequent Klüver-Barrera (KB) staining of the same section followed by similar digitalization enabled to compare the neuronal morphology (KB-stained image) and the epitope presentation (multifluorolabeled image), quantifiable on each neuron throughout the section. This novel strategy, correlative morphometry, appeared very useful to evaluate how quantified indices were correlated with each other on a cell to cell basis. Through comprehensive quantification of those morphological indices in the second sacral segment from two groups of patients with ALS deceased early or late after the onset, it was possible to compare the differences of morphology and neuronal counts in each; 1) region (ON, dorsolateral alpha-motoneuron group and the other areas), 2) phase (early and advanced), and to assess possible influences of TDP43-positive inclusions to these changes. This cell to cell comparison at different time points and in different neuronal groups clarifies what are consistent and fundamental among the morphological changes found in human ALS samples as the first step for understanding the selective neuronal vulnerability of ALS.

## Methods

A sample from the second sacral segment was obtained from eleven patients with sporadic ALS (six early and five advanced cases, each with Bunina bodies) and five control patients without any neurological complications. This study on human samples was performed following The Code of Ethics of the World Medical Association (Declaration of Helsinki) as approved by Institutional Review Board of Tokyo Metropolitan Institute of Medical Science (No 13-22). ALS was defined as early when the patient had died within 1 year after the onset. ALS was considered as advanced when the patient had survived more than 5 years. Patients with any degree of dementia or history of artificial ventilation were excluded. All the post mortem specimens were stored at the Department of Neuropathology of Aichi Medical University (Additional file 1: Table S1). These samples were routinely fixed in formalin and embedded in paraffin to obtain 5 μm-thick sections. After treatment with Sudan-black to reduce autofluorescence of lipofuscin [7], quadruple-fluorolabeling was performed. After incubation with a mixture of three primary antibodies against non-phosphorylated TDP43 (TDP43, polyclonal, rabbit, 1:3,000, Protein Tech, Chicago, Illinois) [8,9], phosphorylated TDP43 (pTDP43, monoclonal, TIP-PTD-M01, mouse, 1:3,000; Cosmo Bio, Tokyo, Japan) [10] and p62 (polyclonal, guinea pig, 1:3,000, Progen, Heidelberg, Germany) [11], the sections were reacted with fluorolabeled secondary antibodies. The epitope for p62 was visualized with biotinylated anti-guinea pig IgG (1:200, Vector, Burlingame, CA)/streptavidin-Alexa Fluor 647^R^ (1:200, Molecular Probes, Eugene, OR) [12]. The epitope for TDP43 was visualized with an anti-rabbit IgG conjugated with Alexa Fluor 546^R^ (1:200, Molecular Probes, Eugene, OR) and that for pTDP43 with an anti-mouse IgG conjugated with Alexa Fluor 488^R^ (1:200, Molecular Probes, Eugene, OR). The nuclei were visualized with DAPI (4′, 6-diamino-2-phenylindole, dihydrochloride, 1:200, Molecular Probes, Eugene, Oregon) for 15 minutes. Fluorescent scanning microscopy (A model equivalent to Pannoramic scan, 3D-Histech, Hungary) enabled to obtain a high-resolution (0.2325 μm/pixel) image of each fluorescence signal tiled to encompass the entire specimen up to 25 × 75 mm (1.08 × 10^5^ × 3.24 × 10^5^ pixels), recorded as digital data. After digital recording of the fluorescent signals, coverslips were removed and the same slides were subjected to KB stain to yield the bright-field counterpart of the fluorescence images, similarly scanned by the same equipment. Once the quadruple fluorescence images had been captured as digital data, identification of all fluoropositive lesions throughout the anterior horn (AH) was possible on the corresponding software, which displays whatever region of interest at whatever magnification (Pannoramic viewer, 3D-Histech, Hungary). Its direct comparison with the KB counterpart (Pannoramic viewer) ensured the precise localization of each fluorescent lesion throughout the AH.

**Figure 1:**
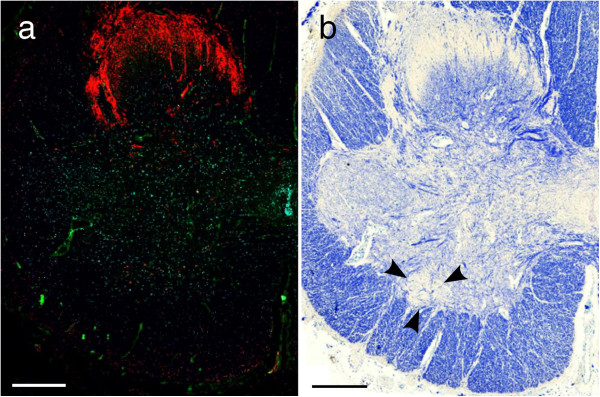
**The Onuf's nucleus (ON) not readily identifiable on immunofluorostained section (a) but clearly identifiable on the Klüver-Barrera (KB)-stained counterpart (b, arrowheads).** Bars = 500 μm.

**Figure 2:**
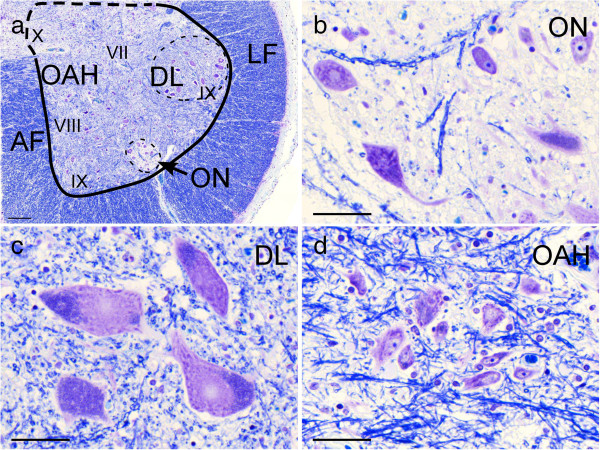
**The three regions of interest and its location on a KB-stained section from a control patient.** Sacral anterior horn in low-power view **(a)**, and high-power views of the ON **(b)**, dorsolateral alpha-motoneuron group of layer IX (DL) **(c)**, and other anterior horn areas (including layer VII, VIII, IX, and X)(OAH) **(d)**. AF: anterior funiculus, LF: lateral funiculus. Bars = **(a)** 200 μm, **(b-d)** 50 μm.

**Figure 3:**
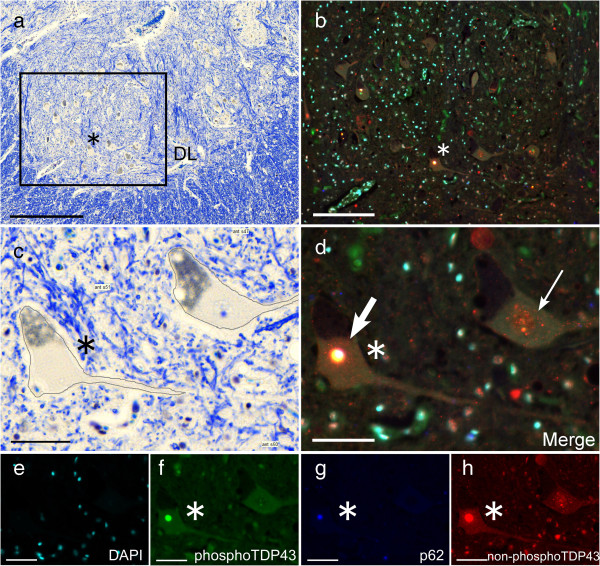
**Parallel identification of the same neurons in DL stained with KB and immunostaining. (a)** Low-power view of the dorsolateral region of sacral spinal cord on KB, and **(b)** immunostaining of the rectangle area in **(a)**. High-power view of this region on KB which enable us to manually trace neurons **(c)** and immunostaining **(d)**. Rounded inclusion (thick arrow) and granular/dot-like deposits (thin arrow) shown in **(d)** which is a merge composed of DAPI (light blue) **(e)**, phosphorylated TDP43 (green) **(f)**, p62 (blue) **(g)** and non-phosphorylated TDP43 (red) **(h)**. Rounded inclusion positive for non-phosphorylated TDP43, phosphorylated TDP43 and p62 **(f, g, h)**. Granular deposits only positive for non-phosphorylated TDP43 **(h)**. The same neuron, indicated by asterisk (*), harboring a rounded inclusion in **(a-d, f-h)**. Bars = **(a)** 500 μm, **(b)** 200 μm, **(c-h)** 50 μm.

Identification of the ON was extremely difficult on the fluorescence-based image (Figure 1a). However, it was readily identified on KB sections as a round-to-oval, sharply demarcated region with scant myelinated fibers located at the ventral margin of AH even in ALS patients (Figure 1b, Figure 2a, Figure 2b). In addition to ON, a group of large neurons with multipolar shape and abundant Nissl bodies and lipofuscin was easily identified in the sacral anterior horn. This group of neurons are usually located and well-developed in dorsolateral part (DL), equivalent to layer IX of Rexed [13,14] of the second sacral segment (Figure 2a). They are representative of the alpha-motor neuron pool (Figure 2a,c). Because precise boundary between other subgroups of the AH neurons was not always evident, neurons outside the ON and DL were grouped as “other AH” (OAH, Figure 2a,d). This OAH group probably contains neuronal subgroups of layer VII (major part of this area), VIII, ventromedial column of IX and X. Mutual reference between KB and fluorescence images allowed to classify each neuron according to its 1) precise localization (ON, DL and OAH on digital KB images), 2) staining profile and 3) cytopathology (skein-like/rounded inclusions or cytoplasmic granular/dot-like deposits). In addition, the transected contour of each neuronal cytoplasm including visible dendrites on KB images was traced manually on the same software (Pannoramic viewer) to automatically calculate the perimeter (μm) and area (μm^2^) of each neuronal cell body in which Nissl granules or lipofuscin, as features of neurons, could be visible in KB (Figure 2b-d, Figure 3c). By screening the entire anterior horn (bilaterally) at the level of the ON, we could gather information of all neurons concerning 1) its location (DL, OAH, and ON), 2) its size (perimeter, area and circularity index (CI) quantifying the morphology of each neuron), 3) the presence of p62/pTDP43 inclusion and 4) the type of inclusions, if present (Figure 3). The types of neurons were classified into three groups according to their size as follows; large (area ≧ 1500 μm^2^), medium (500 ≦ area < 1500 μm^2^) and small (area < 500 μm^2^) [14-16]. The CI was defined by the ratio of two parameters: perimeter/2π (=the radius in case of a circle) and the square root of (area/π) which is also the radius in case of a circle. The ratio is thus 1 in case of a circle. Its value is higher than 1 for more elongated or more blanched forms. It is increased when the neuronal contour is irregular with abundant appendages, mainly dendrites in case of neuronal cytoplasm [17].

Statistical analyses made use of one-way ANOVA and post hoc Scheffe’s multicomparison test (Ekuseru-Toukei 2012, Social Survey Research Information Co., Ltd. Tokyo, Japan) to look for possible differences in neuronal counts and cytological changes (area, perimeter, CI) between stages (control-early-advanced), locations (DL-OAH-ON) and cytopathology (control-with inclusion-without inclusion). The translocation of TDP43 from the nucleus was also analyzed for possible differences between neurons with granular/dot-like deposits and skein-like/rounded inclusions, and between neurons at the early and the advanced phase. Correlation between neuronal count and area or CI was assessed by Spearman’s rank correlation coefficient. A p value <0.05 was regarded as statistically significant.

## Results

### Relative preservation of ON neuron even at the advanced phase

**Figure 4:**
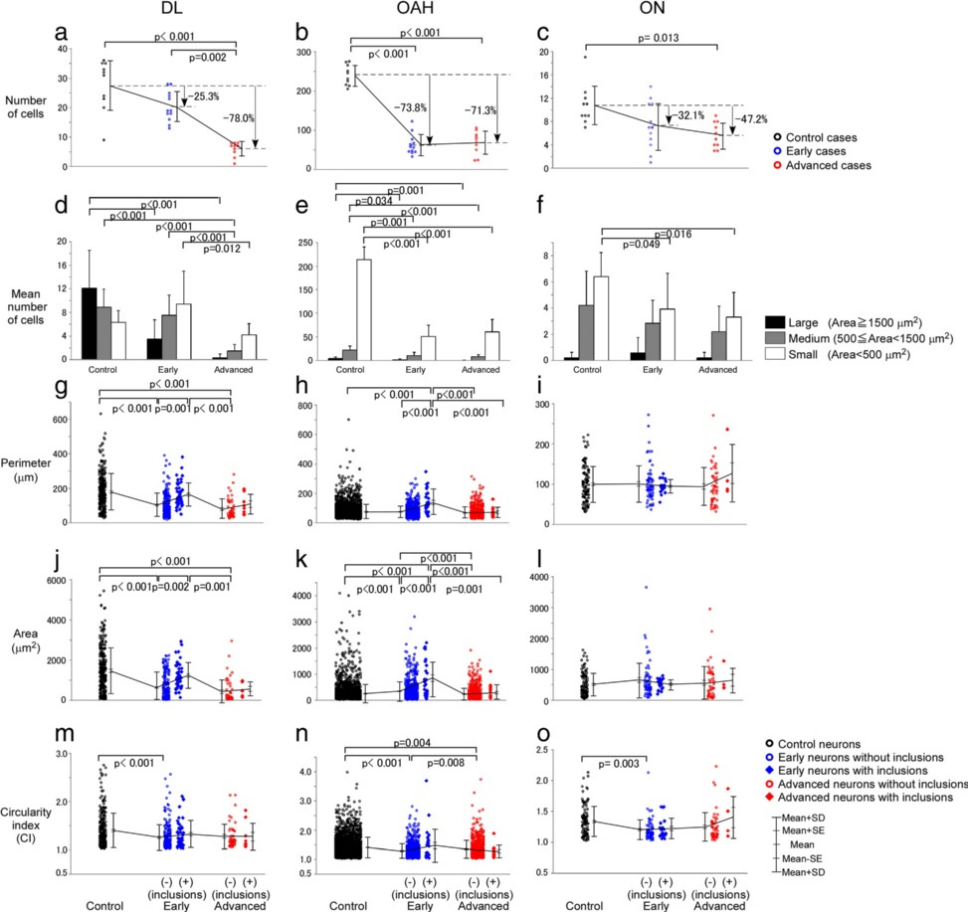
**Progressive loss and cytological changes are attenuated in ON neurons.** Neuronal loss in OAH **(b)** was already severe (-78.8%) at the early phase, while it was less severe in DL (-25.8%) with subsequent progression to -78.0% at the advanced stage. Early loss (-32.1%) and subsequent progression (-47.2%) were attenuated in ON (Figure 3**c**). in DL **(a)** -but mildly to moderately in ON **(c)**. Neuronal loss preferentially involved large neurons in DL **(d)** and small neurons in OAH and ON **(e, f)**. Size reduction of surviving neurons was apparent in DL **(g, j)**, while their size increase was significant in OAH at the early phase **(h, k)**, partly explained by preferential loss of small neurons **(e)**. Neuronal size of surviving ON neurons was stable **(i, l)**. In spite of this variability in size change at the early phase, significant decrease in CI was a consistent feature shared between these groups **(m-o)**. (-): neurons without inclusions, (+): neurons with inclusions. p: p value.

The most severe neuronal loss was observed in OAH. It reached 73.8% (F value (F) =73.6, degree of freedom (df) = 2, p < 0.001) in the early phase, but did not progress in the advanced phase (Figure 4b, Additional file 1: Table S1). This initial drop in the number of neurons followed by a plateau was shared among the different size groups (large, medium and small) of OAH neurons (Figure 4e, Additional file 2: Table S2). Although loss of DL neurons (-25.3%) in the early phase was milder than in OAH, it reached the same level in advanced phase (-78.0%, F = 21.0, df = 2, p < 0.001, Figure 4a). Loss of ON neurons, not significant in the early phase, reached a significant level only at the advanced phase (-47.2%, F = 6.5, df = 2, p = 0.013, Figure 4c).

### Preserved perimeter and area of surviving ON neurons even at the advanced phase

The large and medium neurons of DL decreased in number with progression of illness (Figure 4d, Additional file 2: Table S2). Mean perimeter (Figure 4g) and area (Figure 4j) also decreased in early and advanced phases (Additional file 3: Table S3). In OAH, small neurons, a majority in this group, was markedly decreased already in the early phase, which is at variance with the sparing of small neurons in DL (Figure 4e). Mean area of surviving neurons in OAH at the early phase was larger than that of control (p < 0.001) and returned to the control range in the advanced phase (p < 0.001) (Figure 4k, F = 21.2, df = 4 for a comparison between the 5 groups -control, early inclusion negative, early inclusion positive, advanced inclusion negative, and advanced inclusion positive-, Additional file 3: Table S3). This transient increase in the mean area of OAH neurons at the early phase was not accompanied by the parallel increase in their perimeter (Figure 4h), while the mean perimeter of OAH neurons at the advanced phase was not decreased relative to controls (Figure 4h). The perimeter (Figure 4i) and area (Figure 4l) of ON neurons were stable throughout the early and advanced phase without significant differences relative to controls. The number of large neurons in the ON did not change although those of small neurons decreased (Figure 4f, Additional file 2: Table S2).

### Retraction of dendrites at the early phase in ON as well as in DL and OAH

The CI decreases in parallel with retraction of neuronal appendages (dendrites) or with rounding of neurons. It decreased at the early phase not only in DL (Figure 4m, F = 6.7, df = 4, p < 0.001, Additional file 3: Table S3), where the mean area of neurons also decreased (Figure 4j), but also in OAH (Figure 4n, F = 21.2, df = 4, p < 0.001), where the mean area of neurons increased (Figure 4k). The CI also decreased at the early phase in ON (Figure 4o, F = 5.0, df = 4, p = 0.003), where the mean area remained stable (Figure 4l). Its subsequent change at the advanced phase was limited to OAH neurons, where it was elevated (Figure 4n, F = 21.2, df = 4, p = 0.008).

### Inclusion-associated increase in neuron size in DL and OAH was absent in ON

Inclusion formation in the ON was observed in both early and advanced phase as well as in the DL and OAH. In DL and OAH at the early phase, the perimeters and areas of neurons with inclusions were larger than those without inclusions (DL perimeter: Figure 4g, F = 31.6, df = 4, p = 0.001; DL area: Figure 4j, F = 33.2, df = 4, p = 0.002; OAH perimeter: Figure 4h, F = 15.1, df = 4, p < 0.001; OAH area: Figure 4k, F = 26.2, df = 4, p < 0.001). In the ON, however, these were not significantly different between ALS patients and controls regardless of the presence of inclusions both at the early and advanced phases. The presence of inclusions had no influence on the CI, in all regions (Figure 4m-o).

**Figure 5:**
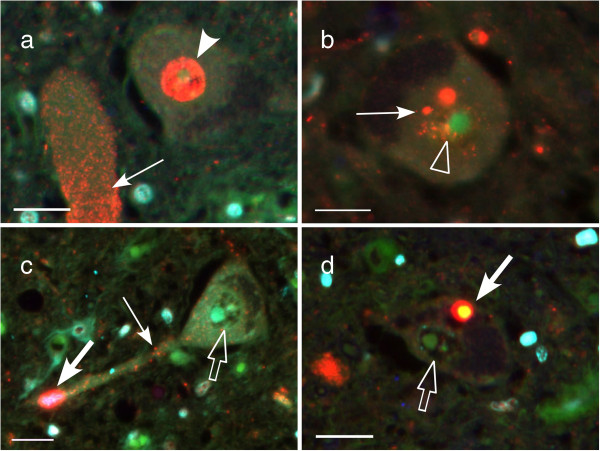
**Granular/dot-like deposits and skein-like/rounded inclusions, associated with loss of nuclear TDP.** Diffuse staining pattern of TDP43 in the nucleus **(a) **(arrowhead), suggestive of intranuclear native TDP43. The granular/dot-like deposits scattered into the cytoplasm (thin arrow) **(a-c)** and nucleus (empty arrowhead) **(b)**. The rounded inclusions in dendrites **(c)** and cytoplasm **(d) **(thick arrows). The nuclear TDP43 hardly detectable in the neurons with rounded inclusions (empty arrows) **(c, d)**. Bars = 20 μm.

**Figure 6:**
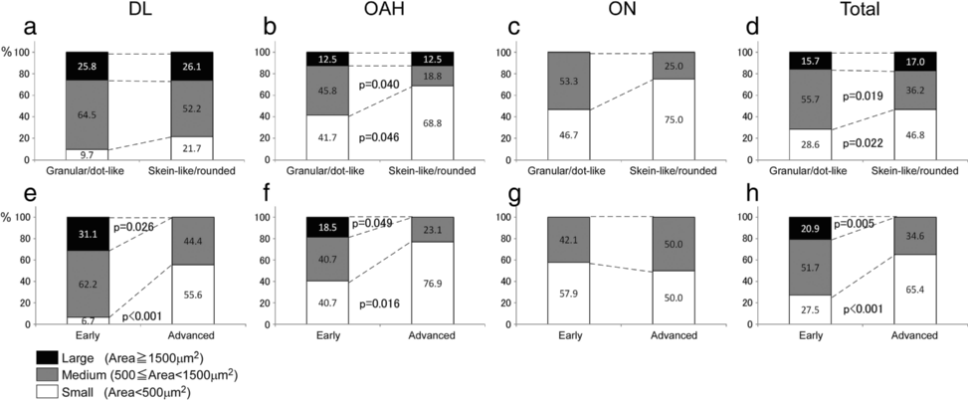
**Progressive loss of large neurons and reciprocal increase in small neurons with skein-like/rounded inclusions.** Skein-like/rounded inclusions more often appear in the small-sized neurons than granular/dot-like deposits, especially in OAH and total areas **(a-d)**. This relative prevalence was increased up to 68.8% in OAH **(b)** upon relative increase of small neurons in OAH (**f**, 76.9%) and DL (**e**, 55.6%) at the advanced stage. A shift in size was absent in ON **(g)**. The relative prevalence of small neurons in total areas was increased up to 65.4% and that of large neurons decreased to 0% at advanced phase **(h)**. Significant p values of differences in prevalence are depicted.

The inclusions were divided into two types according to their shapes (Figure 3d-h). The granular/dot-like deposits, mainly with small granules of non-phosphorylated TDP43, spread over the entire cytoplasm to dendrites (Figure 5a-c; thin arrows). Native (normal) nuclear TDP43 immunoreactivity was completely lost in about one third of neurons with deposits of this type (34.8%, Figure 5c,d; empty arrows, Additional file 4: Table S4). Some TDP43 deposits remaining in the nucleus (Figure 5b; empty arrowhead, Additional file 4: Table S4) were different from the diffuse TDP43 immunoreactivity in normal nucleus (Figure 5a; arrowhead). All skein-like/rounded inclusions were composed of p-TDP43; nuclear TDP43 was lost in most neurons with inclusions of this type (90.9%). The translocation of TDP43 from nucleus to cytoplasm was more frequent in neurons with skein-like/rounded inclusion than those with granular/dot-like deposits (Z = -3.1, p = 0.002, Figure 5c,d; empty arrows, Additional file 4: Table S4), while it was not significantly different between early and advanced phases (Z = 1.0, p = 0.317, Additional file 4: Table S4). Aggregates of p62 often coexisted with p-TDP43 in the skein-like/rounded inclusion (Figure 3g). The prevalence of granular/dot-like deposits and that of skein-like/rounded inclusions were similar in large neurons in DL, OAH and in neurons in ON (Figure 6a-d, Additional file 5: Table S5). The granular/dot-like deposits was more frequent than skein-like/rounded inclusions in medium-sized OAH neurons (45.8% → 18.8%: Z = 1.8, p = 0.040, gray column in Figure 6b) but less frequent in small-sized OAH neurons (41.7% → 68.8%: Z = -1.7, p = 0.046, white column in Figure 6b) to yield similar overall tendency in total (Figure 6d). Progressive decrease in large neuron fraction and reciprocal increase in small neuron fraction was shared between DL (31.1% → 0% (Z = 1.9, p = 0.026) and 6.7% → 55.6% (Z = -3.8, p < 0.001), Figure 6e) and OAH (18.5% →0% (Z = 1.7, p = 0.049) and 40.7% → 76.9% (Z = -2.1, p = 0.016), Figure 6f). Such a shift in size of surviving neurons was absent in ON (Figure 6g).

**Figure 7:**
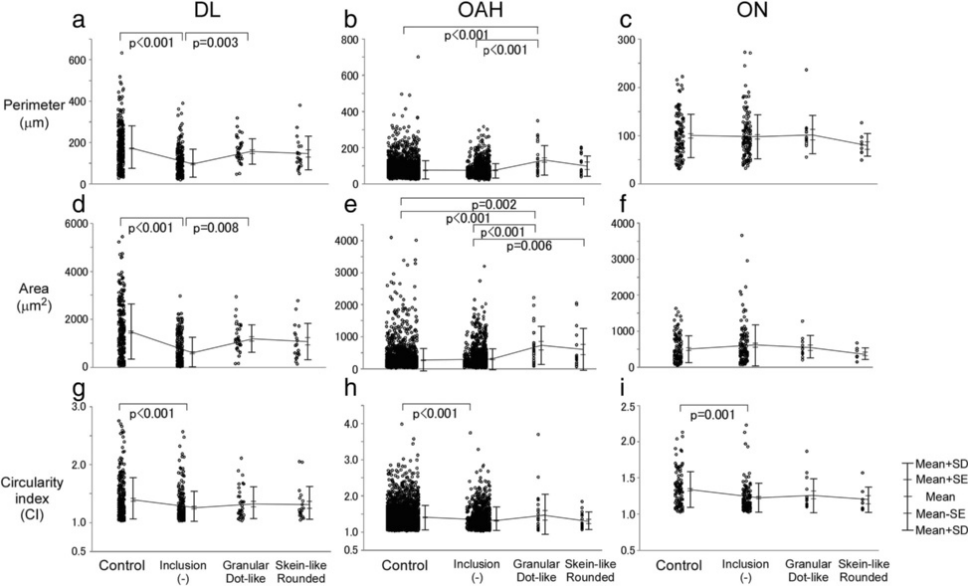
**Size increase with granular-dot like inclusions in OAH neurons and atrophic DL neurons but not in ON.** In DL and OAH, size of neuron without inclusion was smaller than that of granular/dot-like deposits (perimeter: DL; p = 0.003, OAH; p < 0.001)(area: DL; p = 0.008, OAH; p < 0.001) **(a, b, d, e)**. In OAH and ON, neuronal size remained unchanged between control and without inclusion **(b, c, e, f)**. A decrease in CI of neurons without inclusion was consistent in all groups (DL; p < 0.001, OAH; p < 0.001, ON; p = 0.001) **(g-i)**. p: p value.

The values of the perimeter, area and CI were not statistically different between neurons harboring granular/dot-like deposits and those harboring skein-like/rounded inclusions (Figure 7). Perimeter and area were larger in neurons with granular/dot-like deposits than in neurons without, both in the DL (Figure 7a, F = 39.2, df = 3, p = 0.003, Figure 7d, F = 41.1, df = 3, p = 0.008, Additional file 6: Table S6) and OAH (Figure 7b, F = 13.5, df = 3, p < 0.001, Figure 7e, F = 20.0, df = 3, p < 0.001, Additional file 6: Table S6). In ON, perimeter and area were not different in neurons with or in neurons without inclusions (Figure 7c, f, Additional file 6: Table S6). The values of the CI were statistically different between neurons of control and those without inclusions in all regions (Figure 7g, F = 8.65, df = 3, p < 0.001, Figure 7h, F = 23.4, df = 3, p < 0.001, Figure 7i, F = 5.78, df = 3, p = 0.001, Additional file 6: Table S6).

### Retraction of dendrites as a consistent trend along neuronal loss

**Figure 8:**
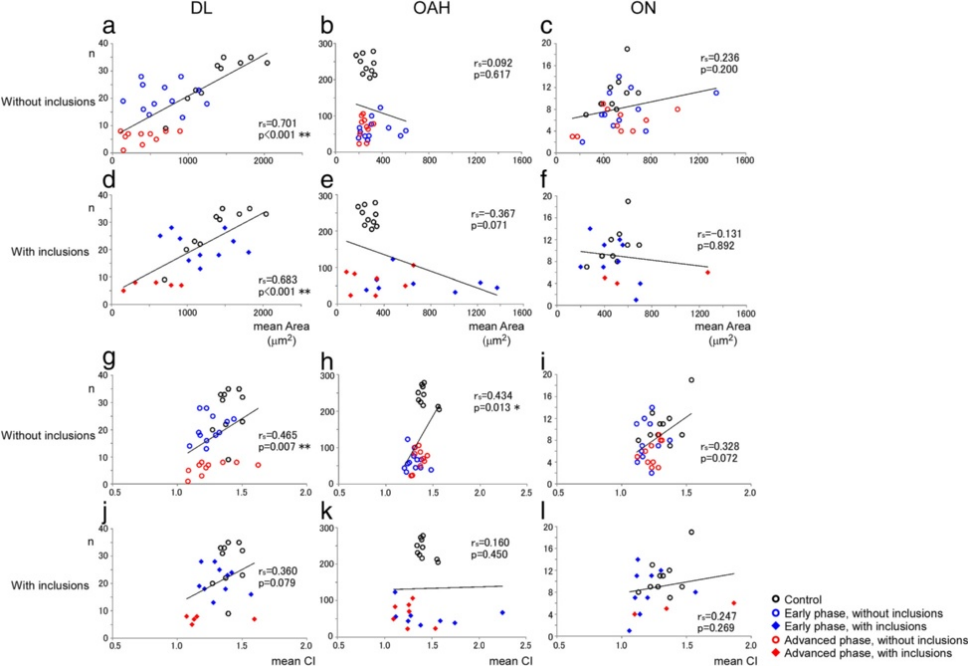
**Decrease of CI was consistent along neuronal loss, but atrophy was not.** In DL, neuronal count parallel with neuronal area (**a**, **d**, p < 0.001). In OAH **(b, e)** and ON **(c, f)**, no parallel relationship between neuronal count and neuronal area (OAH: rs = 0.092, p = 0.617, ON: rs = 0.236, p = 0.200). The CI was correlated with neuronal count in DL **(g)** and OAH **(h)** with statistical significance (DL: rs = 0.465, p = 0.007, OAH: rs = 0.434, p = 0.013). This trend was shared with ON neurons without statistical significance **(i) **(rs = 0.328, p = 0.072). No parallel relationship between neuronal count and CI was apparent in neurons with inclusions in all regions **(j, k, l)**. n: neuronal count, black: control patient, blue: early ALS patient, red: advanced ALS patient p: p value, rs: Spearman’s rank correlation coefficient. *: p value < 0.05, **: p value < 0.01.

A significant positive correlation was observed in the DL, between area decrement and loss of neurons, with (Figure 8d; group with inclusions: rs = 0.683, p < 0.001, Additional file 7: Table S7) or without inclusions (Figure 8a; group without inclusions: rs = 0.701, p < 0.001). The correlation was negative, on the contrary in OAH neurons (especially those with inclusions): their mean area increased while their numerical density decreased (Figure 8e, Additional file 7: Table S7). In ON, neuronal count and area were not significantly correlated (Figure 8f, Additional file 7: Table S7). In contrast, correlation between the count of neurons without inclusions and CI was positive regardless of the neuronal groups (Figures 8g-i), as seen in DL (Figure 8g, rs = 0.465, p = 0.007) and OAH neurons (Figure 8h, rs = 0.434, p = 0.013). A similar trend was observed in ON neurons without inclusions and in DL neurons with inclusions (Figure 8i, j; but did not reach statistical significance).

## Discussion

In the present study, comparison of different neuronal groups in the sacral segment of ALS patients showed that the intensity and the progression of the neuronal loss were both attenuated in ON (-47.2%, Figure 4c) relative to DL (-78.0%, Figure 4a) and OAH (-71.3%, Figure 4b). Although this relative preservation of ON neuron is compatible with previous reports [1,18], our novel strategy of correlative morphometry further characterized the specificity of ON neurons in the course of the disease 1) neuronal perimeter and area of surviving ON neurons remained stable even at the advanced phase, 2) their CI decreased at the early phase -a change that we interpret as evidence of dendritic retraction-, and 3) these parameters were not influenced by the presence of inclusions (Figure 4). This is in contrast with the DL and OAH where the presence of an inclusion was associated with an increase in the area. The decrease in CI was associated with a decrease in cell body area in DL. Unexpectedly, surviving OAH neurons exhibited an increase in area, paradoxically correlated with a decrease in CI at the early phase.

Morphometric studies of the ON neurons are still limited probably due to difficulties in identifying a sufficient number of them. Reports, however, mention that normal neurons decrease in number when the number of abnormal neurons increases [3]; the mean area of neuron cell body is normal [19] or decreases [3]. The mean area of neurons is larger when they contain inclusion than when they do not [3]; the shape (especially of dendrites) does not change [19]. Inclusions (Bunina bodies, skein-like inclusions and round inclusions) are present in ON neurons [2,5,6]. Most of the previous studies dealt with a single morphological parameter studied in one single sacral section. In the present study, correlations between neuronal count, perimeter and area could be investigated. The correlation between the perimeter, area, and CI of neurons with or without inclusion could be analyzed on a cell to cell basis. Comparison between the parameters collected in ON, DL and OAH allowed to further contrast morphological features of ON.

### Attenuated loss and dendritic retraction without size change characterize ON neurons

This study showed that the number of neurons in DL (large motor neurons group) and OAH (the other anterior horn region predominantly containing small neurons) significantly decreased up to 70-80% already at the early phase of the disease in OAH and at the advanced phase in DL. By contrast, those of the ON decreased at the most by 40-50% [20,21]. Both neuronal loss (neuron counts) and atrophy (area in the present study) characterize neurodegeneration and one could assume that they progress in parallel [20,22,23]. This parallelism was clear-cut in DL (Figure 4), while the area of surviving neurons remained unchanged in ON and even increased in OAH. Measurement of neuronal area in different brain regions (anterior horn of the spinal cord, motor nuclei of brainstem cranial nerves and 5th layer of the precentral gyrus) have demonstrated regional heterogeneity and failed to identify a consistent morphological feature shared by all ALS neurons [23]. Even in a single sacral segment (as studied here), heterogeneity between neuronal groups was evident. However, we identified a decrease in CI, probably related to dendrite retraction, as a consistent early feature even in neuronal groups in which neuronal loss and atrophy were heterogeneous (Figure 4) [24,25]. Because further decrease in CI at the advanced phase was not significant even in DL with progressive loss of neurons, dendritic retraction may be a process that reaches a plateau (maximal level) at the early phase. Otherwise, further dendritic retraction may lead to neuronal loss, which is not accessible through morphometric approach of surviving neurons. (Figure 4j-o) [19]. Decrease in CI may occur if elongated neuronal cytoplasm becomes closer to the exact circle (rounding). Although it remains to be clarified whether such rounding is also related to neurite retraction or not, similar approach was already successful for evaluating neurite morphology in human samples [19]. At present, under the limitation of method and human samples, it is reasonable to assume that the CI is one of the best possible and practically feasible indices for neuronal appendages of small number of neurons such as ON.

### The mean area of ON neurons’ cell body did not increase in the presence of inclusions

Although inclusions are hallmarks of many neurodegenerative diseases, it still remains to be settled how they influence the cell survival and morphology [26-28]. With our novel strategy of correlative morphometry, we could show that the inclusion-associated increase in neuronal area was a feature shared by DL and OAH groups (Figure 4). In our previous study, the presence of nuclear inclusion in pontine neurons of Machado-Joseph disease was associated with an increase in nuclear area relative to atrophic neurons without inclusion, suggesting anti-atrophic influence of nuclear inclusions [17,29,30], as demonstrated in DL (Figure 4) or a previous report on ALS [3]. In early OAH, however, the mean area of neurons without inclusions was larger than in controls. Because an additional increase was associated with inclusions, it is probable that inclusions of OAH neurons are related to a kind of hypertrophic (anti-atrophic) process, as we reported it with intranuclear inclusion body disease [29]. Similar increase in area in the OAH neurons without inclusions may represent an intrinsic feature of OAH neurons, not necessarily linked to the presence of inclusion. It is also possible that small inclusions, not detectable on the two-dimensional plane of the section, are however present in another plane and still exert such a hypertrophic effect. Such a possibility could only be confirmed by a three-dimensional reconstruction as we reported on glial cytoplasmic inclusions of multiple system atrophy [30]. On the other hand, in ON, the cell body area of neurons with or without inclusions did not differ significantly.

By contrast, the presence of inclusion had no influence on CI in DL, OAH and ON. A previous report suggested that inclusions were preferentially formed in large neurons [31], our present study demonstrated, however, that these were also observed in small and medium neurons (Figure 6, Additional file 5: Table S5).

It remains to be clarified how abnormal aggregation of TDP43 influences degeneration of motor neurons in ALS patients. A recent experiment on developing drosophila reveals that dendritic branching of sensory neurons increases when normal drosophila TDP43 is overexpressed. Loss of TDP43 in a genetic null mutant or through si RNA, as well as its mutation, decreases dendritic branching [32]. C-terminal TDP43 fragments also impairs dendritic growth whereas normal full-length TDP43 rescues the phenotype in neuronal cultures [33]. TDP43 with missense mutations induces dendritic elongation rather than contraction in cultured cell. Suppression of endogenous TDP43 by si RNA induces similar dendritic abnormalities and cell death [34]. Although it remains to be clarified which of the molecular alterations of TDP43 -overexpression, pathological mutation or truncation-, is responsible for the pathological changes observed in ALS patients, it is probable that similar dendritic retraction as supported in the present study in human autopsy samples is fundamental to ALS pathogenesis.

Our multiple immunofluorolabeling demonstrated frequent colocalization of p62 and pTDP43 in rounded inclusions in the cytoplasm, probably representative of advanced stage or inclusion formation (Figure 3b, thick arrow). Granular cytoplasmic deposits lacking pTDP43 and p62 immunoreactivity (Figure 3d,f,g, thin arrow) may represent an earlier stage of inclusion formation. However, there were no statistical differences of perimeter, area and CI between the groups (Figure 7, Additional file 6: Table S6). This putative evolution from granular cytoplasmic inclusions to rounded inclusion is compatible with the more frequent translocation of TDP43 from nucleus to cytoplasm with rounded inclusions than with granular inclusions. Translocation of TDP43 from nucleus was similarly observed in surviving neurons at the early and the advanced stages without significant difference (57.7% vs. 37.5%: p = 0.317, Additional file 4: Table S4). Even though each of these findings has already been partly published [35,36], our multiple immunofluorolabeling allowed to study the correlations between these changes at a cellular level. Recent reports revealed that the mislocalization of TDP43 (loss of nuclear TDP43 and cytoplasmic aggregation of TDP43) may lead to a suppression of mRNA transcripts, abnormal cytoplasmic-nuclear RNA transport, or local translation of mRNA. These disruptions of RNA function possibly induce cellular abnormalities including dendritic retraction possibly leading to motor neuron degeneration as suggested by the present study involving human brain tissue [32,33,37,38].

### Retraction of dendrites as a consistent feature of ALS neurons

A decrease in the mean cell body area (atrophy) is generally believed to be the prelude of neuronal loss [20,22,23]; it was indeed the case in DL (Figure 8a,d). Quite unexpectedly, however, we could not demonstrate such a correlation in ON (Figure 8c,f). The correlation was significant but reversed in OAH (Figure 8b,e). Similarly, a decrease in CI may naturally precede neuronal loss. This was indeed the case for all the neuronal groups (Figures 8g-l) with significant correlation in DL (p = 0.007, Figure 8g) and OAH (p = 0.013, Figure 8h) groups when the neurons without inclusions were considered. The lack of a significant correlation in ON (p = 0.072) may be explained by the rostrocaudal extension of most of the dendrites of ON neurons [39]. Those dendrites have probably no or but a small influence on the CI measured in a transverse plane, as in the present study. Moreover, attenuated neuronal loss and maintained neuronal cell body area in ON might blur this correlation, even though a decrease in CI is significant at the early stage (Figure 4o). It is also possible that morphological changes of ALS neurons are so variable from a neuron to another or from a patient to another to escape or distort expected correlation as seen in some cases with severe neuronal loss but relatively preserved neuronal area (Figures 8e, f) and dendritic arborization (Figures 8k, l). Anyway, the decrease in CI appears to be the most consistent, and therefore fundamental change in ALS than alteration of the area of the neuronal cell body.

## Conclusion

A section from the sacral segment of ALS patients and controls was studied. After triple (p62, TDP43, pTDP43) immunofluorescence and DAPI staining, followed by KB technique, the area, perimeter and CI of each neuron belonging to different neuronal groups (DL, OAH and ON) were measured. The relation between these variables and TDP43 immunoreactivity was investigated. Attenuated neuronal loss and preservation of the neuronal size were characteristic of ON. Presence of TDP43-positive inclusions was associated with an increase in neuron size in DL and OAH, while this influence was not significant in ON. Although neuronal loss, size and influence of TDP43-positive inclusions were variable according to neuronal groups, drop of CI, related to dendritic retraction, was a consistent feature shared by all studied neuronal groups (DL, OAH and ON). The present study demonstrated that dendritic retraction may be a fundamental morphological change underlying ALS pathogenesis. Three-dimensional reconstruction could even increase the observed prevalence of these changes in ALS. Neuronal loss finally appears a late event. Inclusion formation and neurite abnormalities occur much earlier. They, rather than neuronal death, possibly provide a better basis to develop new biomarkers and therapeutic targets.

### Consent

Written informed consent for research use of autopsy materials was obtained from the patient’s guardian/parent/next of kin.

## Competing interests

The authors declare that they have no competing interests.

## Authors’ contributions

TT, TU and YN planned this study. TU, AN and MY prepared and stained histological sections. TT and TU carried out the histological analyses and collated the neuropathological data. TT, TU wrote the manuscript. SS, SK, SU and CD critically read and revised the manuscript. All authors read and approved the final manuscript.

## Supplementary Material

Additional file 1: Table S1 Clinical and morphometric profiles of 11 ALS and 5 control patients. (Footnote DL: dorsolateral alpha-motoneuron group, OAH: other anterior horn neurons, ON: Onuf’s nuclei, CI: circularity index). Click here for file

Additional file 2: Table S2 Numbers of surviving neurons in each size. (Footnote L: Large, M: Medium, S: Small). Click here for file

Additional file 3: Table S3 Chronological and regional differences in neuron morphology with or without inclusions. Click here for file

Additional file 4: Table S4 More frequent TDP43 mislocalization with skein-like/rounded inclusions. (Footnote KB: Klüver-Barrera method. #: prevalence of nuclear loss of TDP43 in neurons carrying cytoplasmic TDP43 deposits). Click here for file

Additional file 5: Table S5 Number of neurons with inclusions in each size of neurons. (Foot note Each percentage is depicted in parenthesis). Click here for file

Additional file 6: Table S6 Influence of inclusions on neuron morphology in different groups. Click here for file

Additional file 7: Table S7 Case-to-case profiles of neurons with/without inclusions. (Footnote N: no neurons with/without inclusions). Click here for file
