# Supplementary figures and images for: Dendritic retraction, but not atrophy, is consistent in amyotrophic lateral sclerosis-comparison between Onuf’s neurons and other sacral motor neurons-
Source: Acta Neuropathol Commun. 2014 Jan 27;2:11. doi: 10.1186/2051-5960-2-11 (PMC3922781; doi:10.1186/2051-5960-2-11)

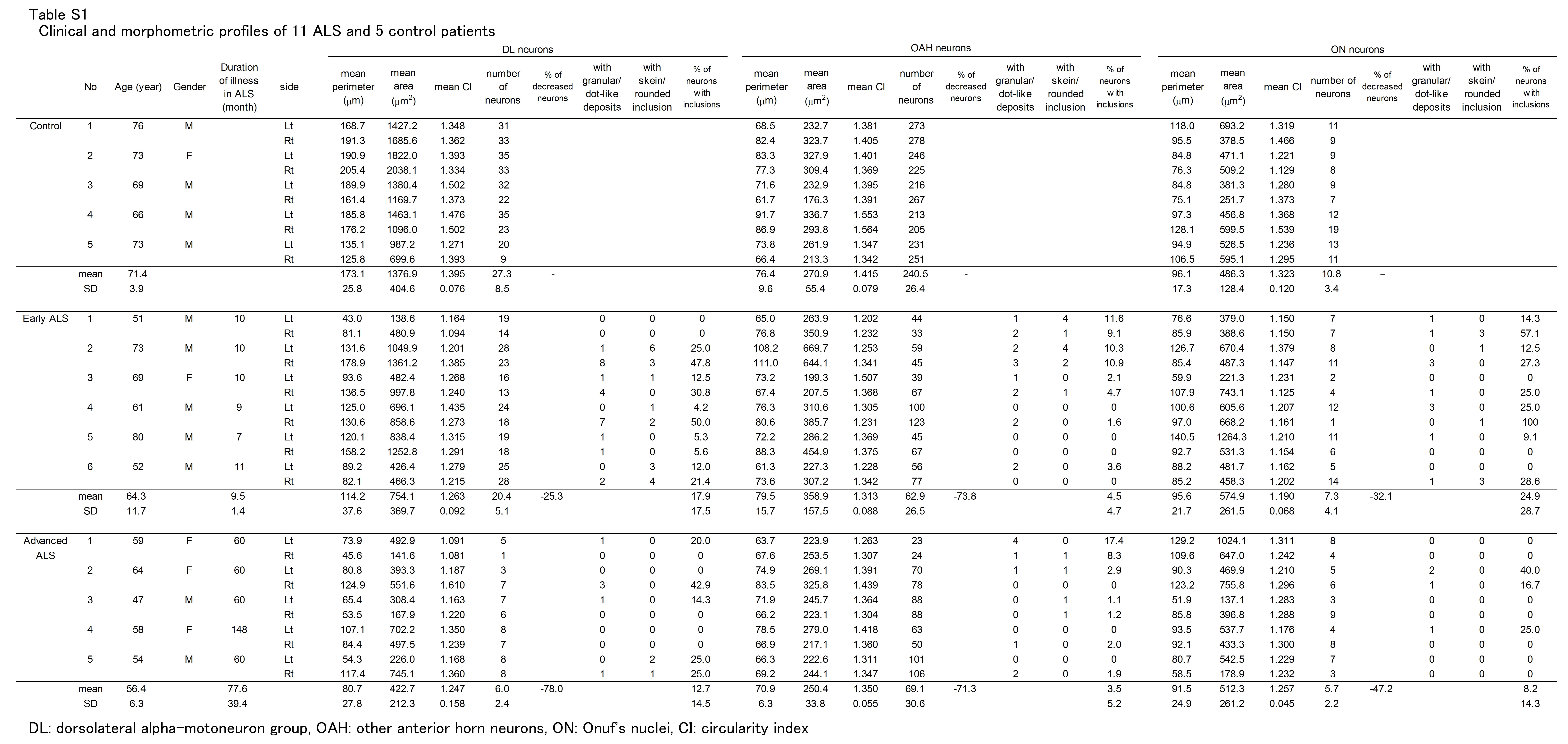

Supplement: Additional file 1: Table S1 — Clinical and morphometric profiles of 11 ALS and 5 control patients. (Footnote DL: dorsolateral alpha-motoneuron group, OAH: other anterior horn neurons, ON: Onuf’s nuclei, CI: circularity index). [file 2051-5960-2-11-S1.jpg]

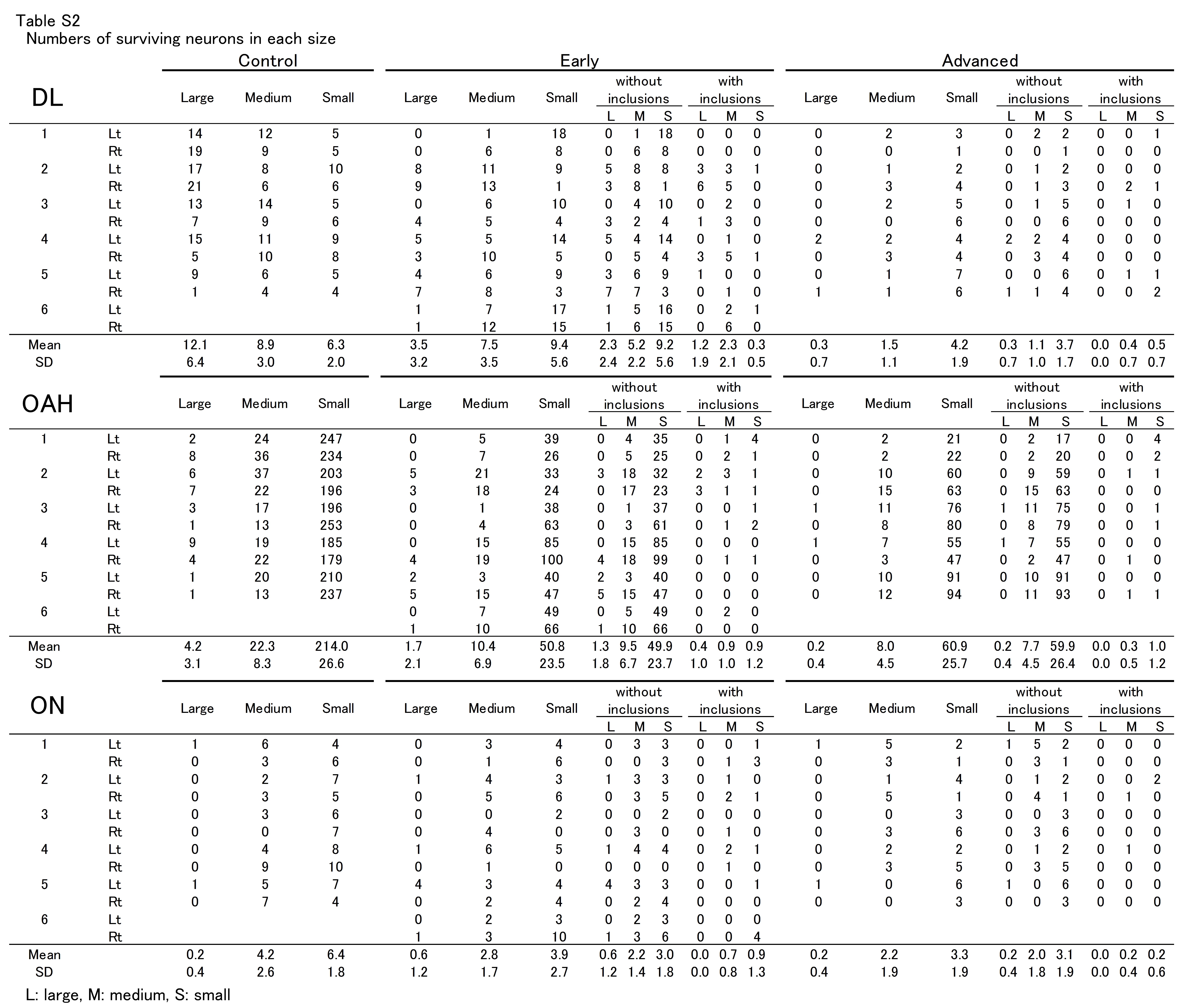

Supplement: Additional file 2: Table S2 — Numbers of surviving neurons in each size. (Footnote L: Large, M: Medium, S: Small). [file 2051-5960-2-11-S2.jpg]

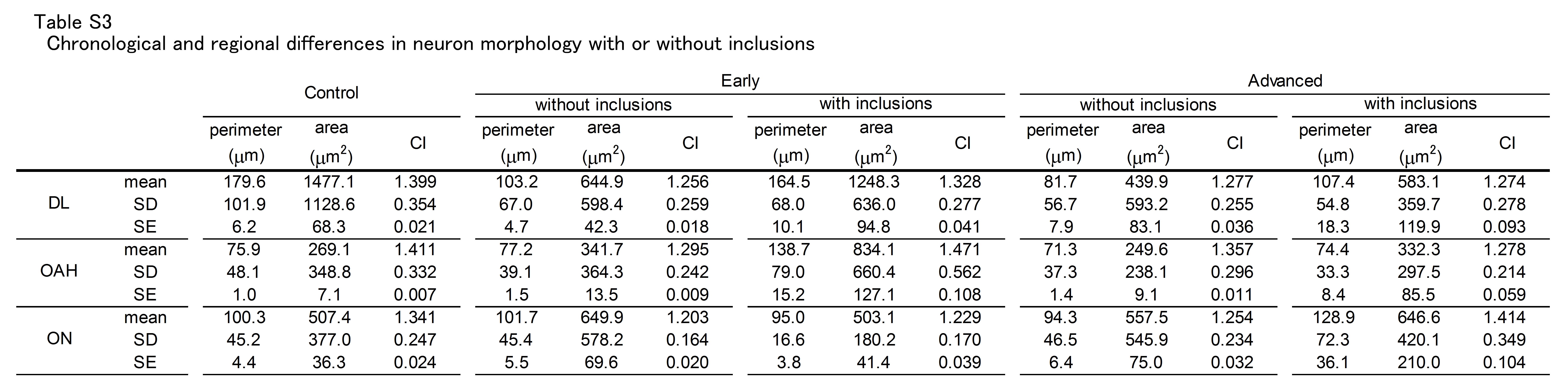

Supplement: Additional file 3: Table S3 — Chronological and regional differences in neuron morphology with or without inclusions. [file 2051-5960-2-11-S3.jpg]

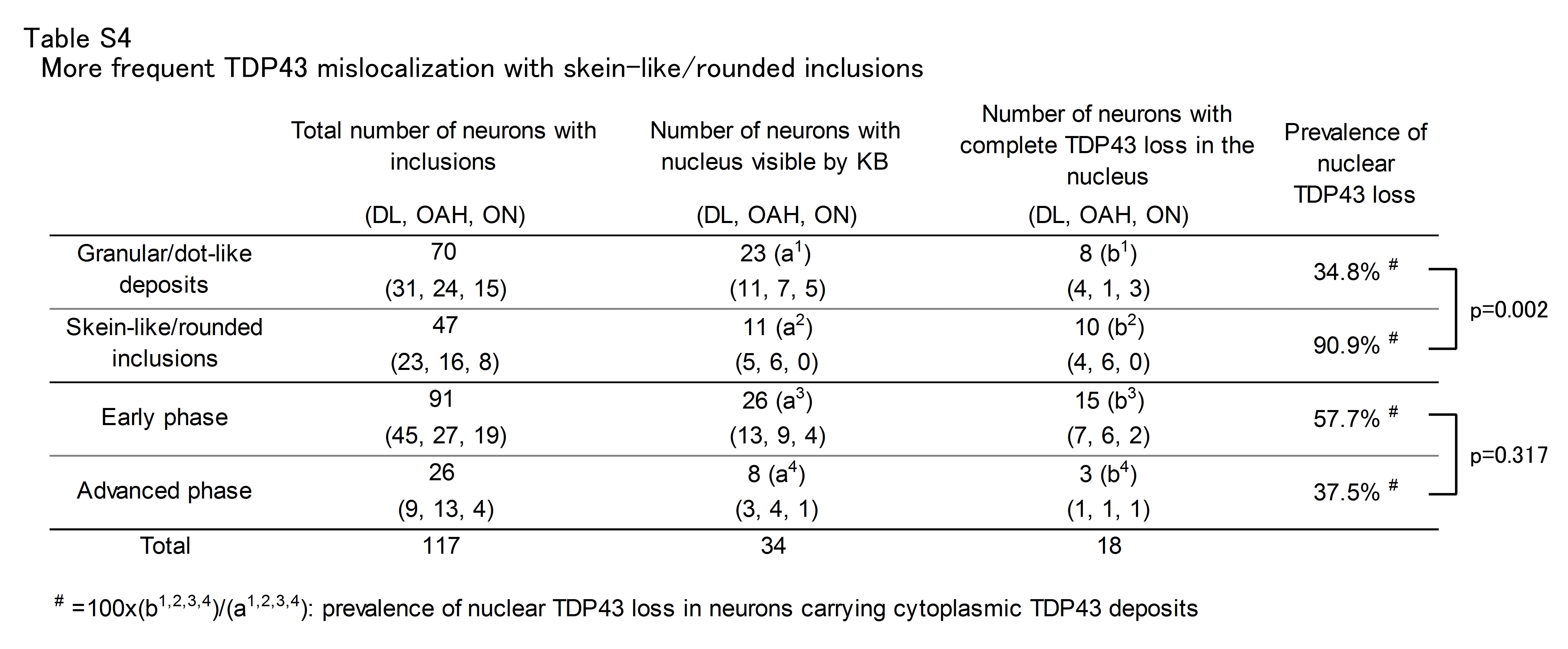

Supplement: Additional file 4: Table S4 — More frequent TDP43 mislocalization with skein-like/rounded inclusions. (Footnote KB: Klüver-Barrera method. #: prevalence of nuclear loss of TDP43 in neurons carrying cytoplasmic TDP43 deposits). [file 2051-5960-2-11-S4.jpg]

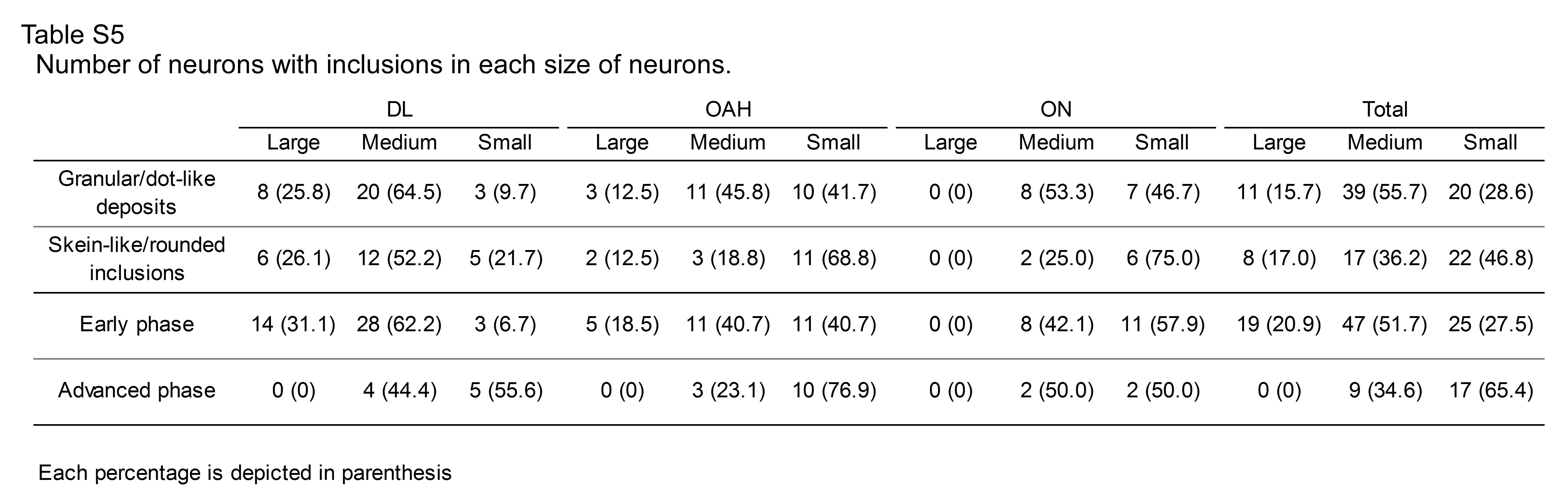

Supplement: Additional file 5: Table S5 — Number of neurons with inclusions in each size of neurons. (Foot note Each percentage is depicted in parenthesis). [file 2051-5960-2-11-S5.jpg]

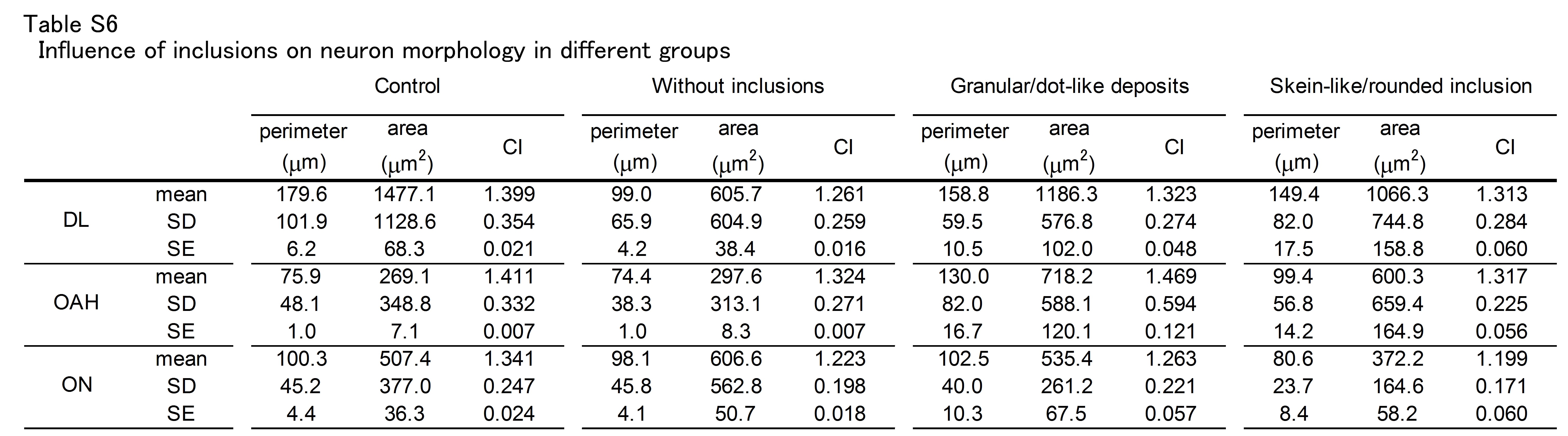

Supplement: Additional file 6: Table S6 — Influence of inclusions on neuron morphology in different groups. [file 2051-5960-2-11-S6.jpg]

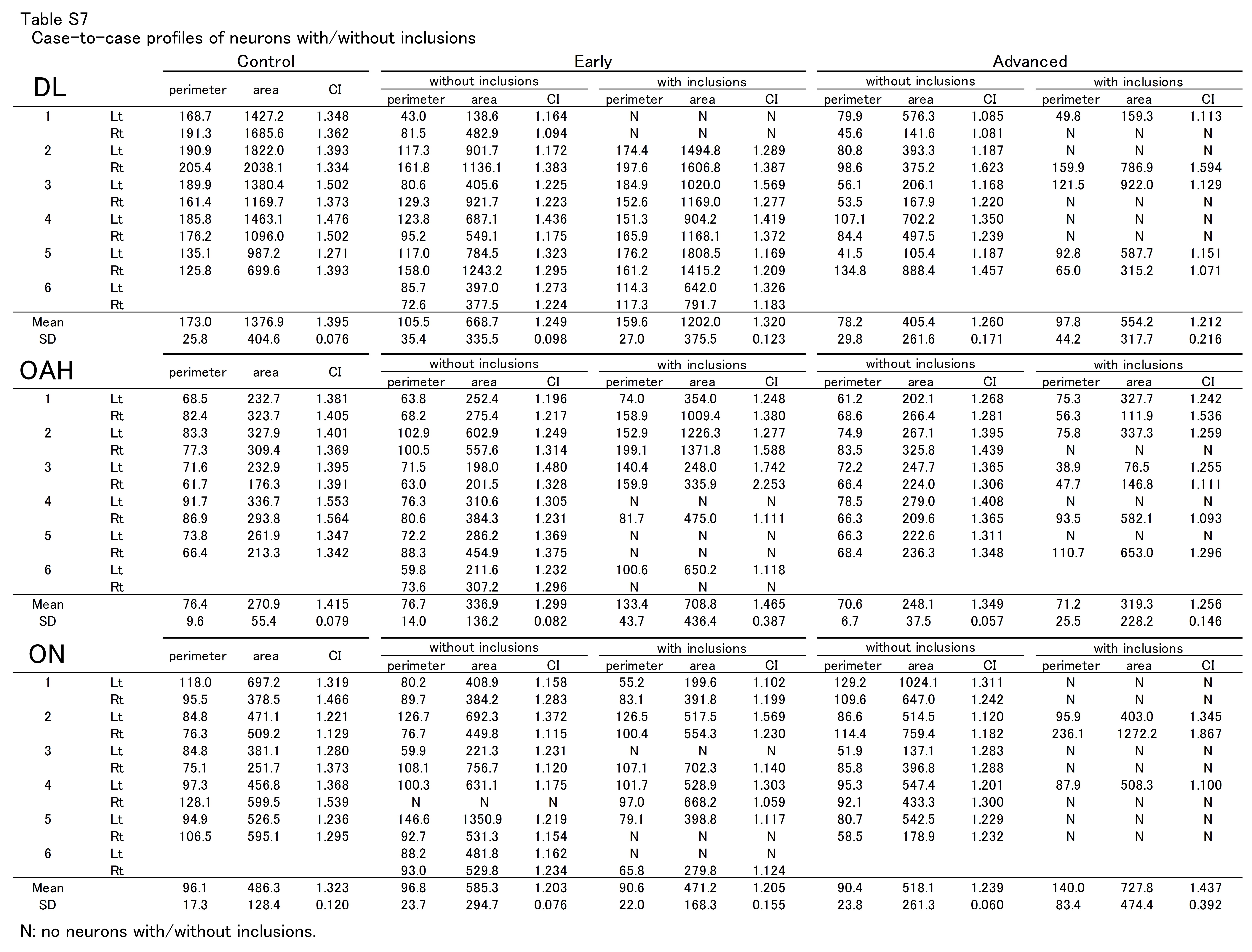

Supplement: Additional file 7: Table S7 — Case-to-case profiles of neurons with/without inclusions. (Footnote N: no neurons with/without inclusions). [file 2051-5960-2-11-S7.jpg]
